# Multicenter validation of a mouse model for cow's milk allergy to assess the allergenicity of hydrolysed cow's milk based infant formulae

**DOI:** 10.1186/2045-7022-3-S3-P142

**Published:** 2013-07-25

**Authors:** BCAM van Esch, JHM Van Bilsen, PV Jeurink, J Garssen, KCM Verhoeckx, JJ Smit, RHH Pieters, LMJ Knippels

**Affiliations:** 1 Utrecht Institute for Pharmaceutical Sciences, Utrecht, the Netherlands; 2 Danone Research Centre for Specialised Nutrition, Wageningen, the Netherlands; 3 TNO, Zeist, the Netherlands; 4 Institute for Risk Assessment Sciences, Utrecht, the Netherlands

## Background

The EC-directive 2006/141/E requires objective and scientifically verified data to claim hypoallergenicity of hydrolysed formulae. However, no validated animal models are currently available to assess the residual sensitising capacity, although guinea pig assays are frequently used. This study is part of a multi-phase project which aims to validate a recently developed mouse model to assess the potential allergenicity of hydrolysed cow's milk based infant formulae. The discriminatory power of a mouse allergy model for cow's milk allergy to distinguish sensitizing properties of cow's milk products was evaluated in 4 independent research centers.

## Methods

C3H/HeOuJ mice were sensitised by oral administration of whey (WPC80) or extensively hydrolysed cow's milk (eWH) at weekly intervals for 5 weeks. One week after the last sensitisation, the acute allergic skin response (ear swelling at 1 hr), anaphylactic symptoms and temperature were determined upon intradermal ear injection of whey. Subsequently, mice were challenged orally with 50 mg whey and blood samples were taken after 30 min. Serum was analyzed for whey-specific immunoglobulins and mMCP-1. All protocols, test substances and procedures were standardised.

## Results

All participating research laboratories detected elevated levels of whey-specific IgE/IgG1/IgG2a, serum mMCP-1 (reflection of mast cell degranulation) and acute allergic skin responses in whey sensitised animals. Anaphylactic symptoms and temperature drop were scored in 3 out of 4 research centers. In contrast, none of the evaluated parameters was altered in eWH-sensitized groups as compared to the vehicle control group.

## Conclusion

All 4 independent research centers were able to discriminate between the sensitising properties of whey protein and extensively hydrolysed cow's milk. The results demonstrate the discriminatory power of the mouse model and thereby the suitability of the mouse model to evaluate the allergenicity of hydrolysed cow's milk formulae. In the next phase of the multicenter validation process, partially hydrolysates will be included to assess the sensitivity of the discriminatory power of the mouse model.

## Disclosure of interest

B van Esch: Employee of Danone Research Centre for Specialised Nutrition, J Van Bilsen: None declared, P Jeurink: Employee of Danone Research Centre for Specialised Nutrition, J Garssen: Employee of Danone Research Centre for Specialised Nutrition, K Verhoeckx: None declared, J Smit: None declared, R Pieters: None declared, L Knippels: Employee of Danone Research Centre for Specialised Nutrition.

